# Research progress of neuroinflammation-related cells in traumatic brain injury: A review

**DOI:** 10.1097/MD.0000000000034009

**Published:** 2023-06-23

**Authors:** Qinghui Zhao, Huige Li, Hongru Li, Fei Xie, Jianhua Zhang

**Affiliations:** a Institute of Physical Culture, Huanghuai University, Zhumadian, China; b Zhumadian Central Hospital, Zhumadian, China; c Faculty of Environment and Life, Beijing University of Technology, Beijing, China

**Keywords:** inflammatory cells, neuroinflammation, traumatic brain injury

## Abstract

Neuroinflammation after traumatic brain injury (TBI) is related to chronic neurodegenerative diseases and is one of the causes of acute secondary injury after TBI. Therefore, it is particularly important to clarify the role of cellular mechanisms in the neuroinflammatory response after TBI. The objective of this article is to understand the involvement of cells during the TBI inflammatory response (for instance, astrocytes, microglia, and oligodendrocytes) and shed light on the recent progress in the stimulation and interaction of granulocytes and lymphocytes, to provide a novel approach for clinical research. We searched articles in PubMed published between 1950 and 2023, using the following keywords: TBI, neuroinflammation, inflammatory cells, neuroprotection, clinical. Articles for inclusion in this paper were finalized based on their novelty, representativeness, and relevance to the main arguments of this review. We found that the neuroinflammatory response after TBI includes the activation of glial cells, the release of inflammatory mediators in the brain, and the recruitment of peripheral immune cells. These inflammatory responses not only induce secondary brain damage, but also have a role in repairing the nervous system to some extent. However, not all of the mechanisms of cell-to-cell interactions have been well studied. After TBI, clinical treatment cannot simply suppress the inflammatory response, and the inflammatory phenotype of patients’ needs to be defined according to their specific conditions after injury. Clinical trials of personalized inflammation regulation therapy for specific patients should be carried out in order to improve the prognosis of patients.

## 1. Introduction

Traumatic brain injury (TBI) is a functional or pathological change in the brain caused by external forces. It is a major public health problem around the world. Studies suggest that post-traumatic stress disorder, memory disorder, Parkinson’s disease, epilepsy, and chronic traumatic encephalopathy are all related to neuroinflammatory response.^[1–5]^ Inflammation is associated with fighting, invading pathogens, and maintaining tissues’ physiological health. However, under pathological conditions such as trauma, inflammation can also aggravate tissue damage.^[6–8]^ Initially, it was believed that TBI’s inflammatory response occurs via impaired blood–brain barrier (BBB) by peripheral immune mediators, however, it was then proved that it occurs through a complex interaction of central, peripheral, and soluble cells, and is controlled by a variety of factors. After TBI, microglia initiate the infiltration of peripheral neutrophils followed by lymphocytes and mononuclear macrophages, while various inflammatory cytokines and chemokines stimulate immune cells and accumulate in the lesion area.^[9–12]^ Inflammatory response after TBI has advantages along with certain disadvantages, such as it promotes debris clearance, neuronal regeneration, glial cell differentiation, and angiogenesis and is harmful as it mediates neuronal death and neurodegenerative diseases.^[5,10,12,13]^ Therefore, a comprehensive understanding of neuroinflammatory responses after TBI is particularly important. This review focuses on the latest research associated with cells related to neuroinflammatory response after TBI and their interactions concerning the 2 aforementioned aspects.

## 2. Inflammatory response and glial cells after TBI

### 2.1. Inflammatory response and microglia initiation after TBI

**Figure 1.**
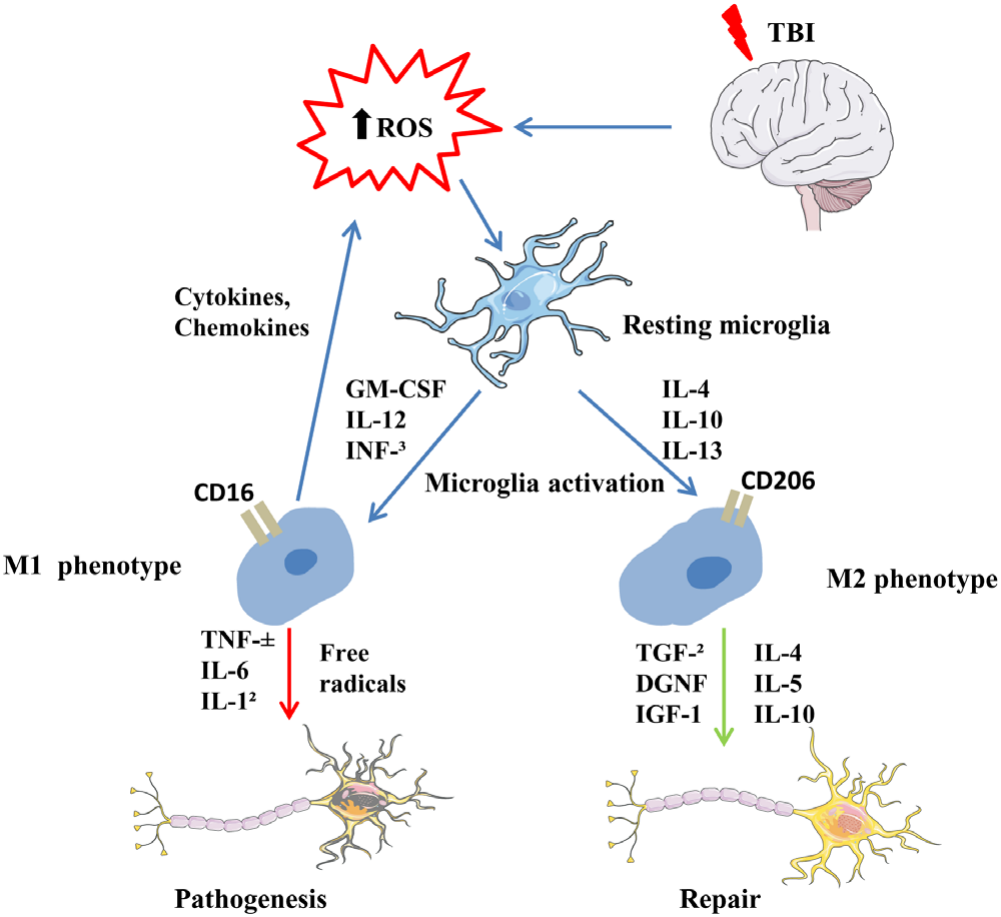
Inflammatory response and microglia initiation after TBI. TBI = traumatic brain injury.

The microglia cells have functions similar to that of peripheral macrophages and are easily affected by the surrounding microenvironment to polarize to different phenotypes. Polarized microglial cells are categorized into the M1 and M2 types, and the M2 is further divided into M2a, M2b, and M2c subtypes.^[13]^ The M1-like phenotype is stimulated mainly by damage-associated molecular patterns (DAMPs), free radicals, or pro-inflammatory cytokines, and is manifested by increased chemokines, pro-inflammatory cell factors, reactive oxygen species production, and decreased cytophagocytosis activity. M1-like “pro-inflammatory” microglia cells are generally considered harmful, and their excess or prolonged responses can cause secondary brain damage. M2a-like microglia produce interleukins interleukin (IL)-4 and IL-13, which are linked with increased anti-inflammatory cytokines production and phagocytosis. M2c-like microglia may promote tissue repair and remodeling. M2b-like can be stimulated by toll-like receptor ligands and has both pro-inflammatory and anti-inflammatory effects (Fig. 1). However, the dominant polarization type of microglia changed with the time after TBI and the different TBI models, and most activated microglia were mixed with M1/M2-like microglia. Studies have shown that M2-type microglia can promote tissue repair and have anti-inflammatory effects, while the abnormal increase of M1-type microglia further advances pathological conditions.^[14]^ Therefore, the early treatment strategy of TBI should not only inhibit the activation of microglia, but also pay more attention to increasing the proportion of M2-type microglia. Recent studies suggest that the following factors can improve the M2/M1 ratio: Atorvastatin, deoxycholic acid taurine, sinomenine, hydrogen inhalation, colony-stimulating factor-1, erythropoietin, TAK-242, Minocycline, Fingolimod, Peroxisome proliferator-activated receptors, Progranulin, Laquinimod, and Fingolimod, Fingormode, estrogen, and progesterone, omega-3 polyunsaturated fatty acids, Histone deacetylase inhibition, suppressors of cytokine signaling, complement components, IL-1 receptor antagonist, stem cells, hypothermia, and Maraviroc; all of these mediators have good anti-inflammatory effects at the level of animal models and play a certain role in neuroprotection after TBI.^[15–22]^ Of course, large amounts of clinical data and further mechanistic studies are still needed in the future.

### 2.2. Inflammatory response and astrocyte initiation after TBI

**Figure 2.**
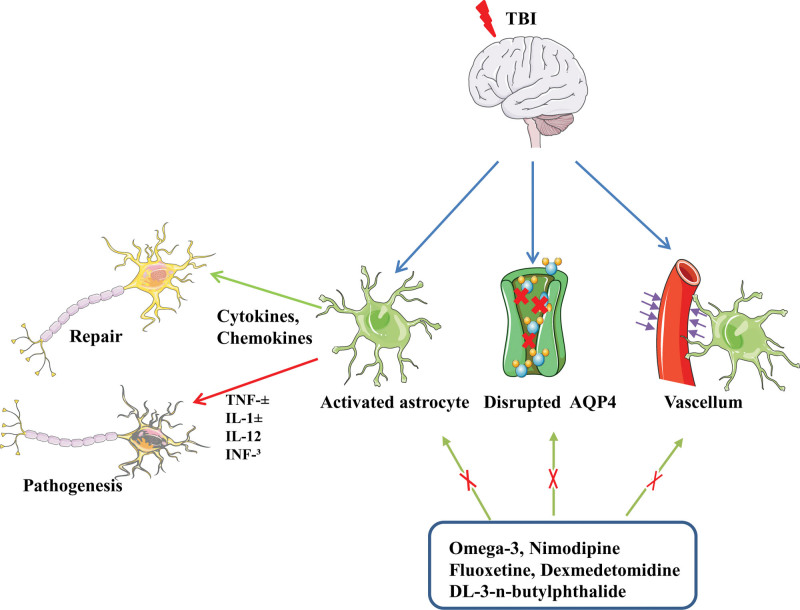
Inflammatory response and astrocyte initiation after TBI. TBI = traumatic brain injury.

Cell swelling is the main pathophysiological mechanism of increased intracranial pressure after TBI and astrocytoma-mediated nuclear factor kappa-B (NF-κB) signaling is responsible for this cell swelling. Therefore, inhibition of NF-κB signaling in astrocytes can reduce the inflammatory response after central nervous system (CNS) injury.^[23–25]^ Small et al^[26]^ found that after TBI, reactive astrocytes could increase the possibility of epilepsy after TBI by inducing JNK signal transduction. Additionally, after TBI, the stimulated high mobility group protein 1 (HMGB1) sends signals to microglia to induce IL-6 secretion and to reactive astrocytes to up-regulate the aquaporin-4 (AQP4) water channel involved in water uptake.^[27]^ The negative side of reactive astrocytes is that they can directly increase intracranial pressure due to cytotoxic edema and produce harmful inflammatory mediators that aggravate brain injury. Although Walko et al^[28]^ found that increasing the concentration of DAMPS, HMGB1 and mitochondrial deoxyribonucleic acid in cerebrospinal fluid (CSF) aggravates the condition of patients with TBI, astrocytic DAMPs can also cause them to interact with phagocytes, thus promoting the clearance of toxic debris.^[29,30]^ Moreover, after the brain injury, the reactive astrocytes release signals such as HMGB1 that stimulate endothelial cells and their progenitors to boost BBB repair and neurovascular remodeling.^[31,32]^ Rosa’s et al^[33]^ study found that toll-like receptor 4-induced astrocyte activation could improve synaptic and cerebrovascular integrity after TBI. In addition, NF-κB signaling in astrocytes may be beneficial after brain injury as it stimulates the production and secretion of brain-derived neurotrophic factor, nerve and glia-protective growth factor, and nerve growth factor.^[30]^ In addition, as a component of the glymphatic system, astrocytes participate in the clearance of metabolites in the brain, and the phalopodium of astrocytes intensively expresses AQP4 water channel, which helps cerebrospinal fluid flow into the brain parenchyma.^[34]^ Ren et al^[35]^ studied the expression and localization of AQP4 in mild and moderate TBI. They found that increased expression of this channel was associated with depolarization. These changes correlated with the proportion of reactive astrocyte hyperplasia. According to Iliff et al,^[36]^ deletion of aquaporin 4 gene leads to increased Tau levels after TBI. This promotes intracellular aggregation of proteins, along with axonal degeneration, neuroinflammation, and worsening of cognitive impairment. Plog and Nedergaard^[37]^ evaluated the clearance rates of TBI-specific markers such as S100b, GFAP and NSE through experiments. The results showed that the change of AQP4 channel reduced the clearance rate of TBI biomarkers. Therefore, targeting reactive gliosis may be a therapeutic strategy to normalize AQP4 expression and improve clearance of interstitial waste after TBI. Zhang et al^[38]^ found that omega-3 intake may be a potential treatment for astrocyte alterations after TBI, as the administration of fish oil for 2 months prior to the determination of TBI significantly improved neuronal function in mouse models, preventing aßs from accumulating by partially restoring the expression and depolarization of TBI-damaged AQP4. In addition, drugs such as Nimodipine, Dexmedetomidine, Selective serotonin reuptake inhibitor: fluoxetine, DL-3-n-butylphthalide can improve the function of glymphatic system and play a neuroprotective role.^[39]^ In summary, at different injury levels and their associated time points, astrocytes produce different inflammatory molecules, immunomodulatory molecules, cytokines, chemokines, and growth factors.^[40–43]^ Therefore, during the investigation of the anti-inflammatory and proinflammatory effects of astrocytes produced by TBI, the influence of the degree and time associated with the injury should be considered (Fig. 2).

### 2.3. Inflammatory response and oligodendrocyte initiation after TBI

**Figure 3.**
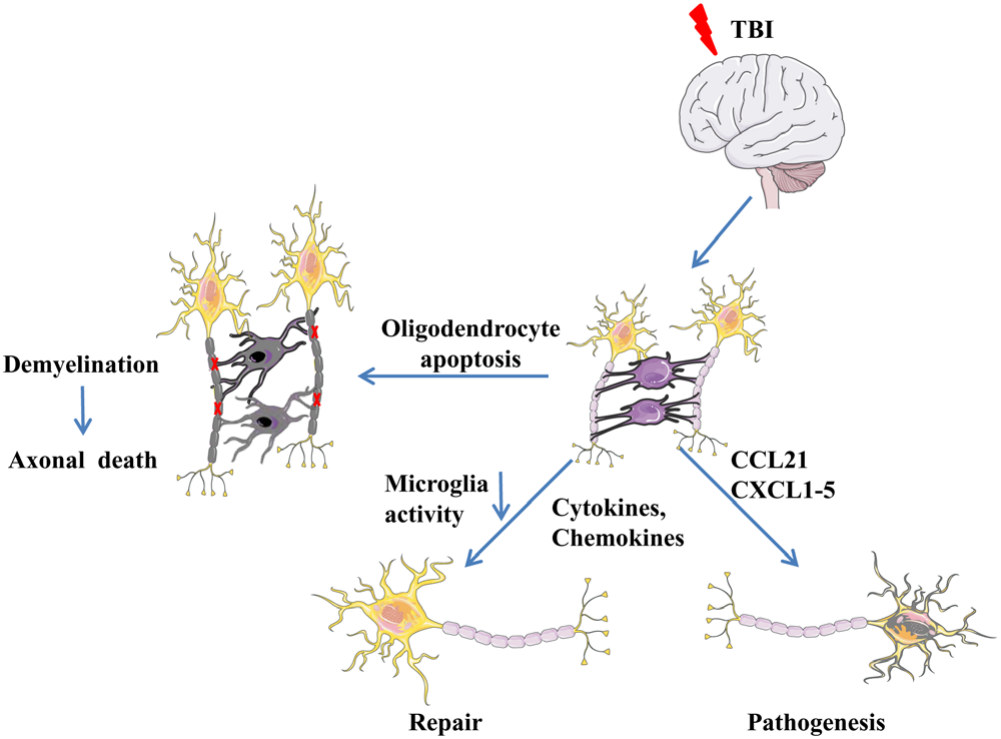
Inflammatory response and oligodendrocyte initiation after TBI. TBI = traumatic brain injury.

Axons and blood vessels are wrapped by mature oligodendrocytes to form a myelin sheath, which serves as nerve insulation and maintains normal neuronal functions. Mature oligodendrocytes are categorized into 3 types based on their distribution: intervascular, perineuronal, and perivascular oligodendrocytes. The intervascular subtype is mainly found along the nerve fibers and white matter and decreases rapidly during the myelin stage. The peri-neuronal subtype, also known as peri-neuronal satellite cells, can be generated from the oligodendrocyte progenitor cells (OPCs) after an injury or pathological condition. Activation of OPCs is considered to be a brain-protective response to regulate homeostasis and assist in post-injury repair.^[43–46]^ After brain injury, oligodendrocytes can act as receptors of neuroinflammatory mediators, such as IL-6, IL-4, IL-7, and chemokine receptors 1, chemokine receptors 2, chemokine receptors 4. Chemokine receptors 4 can bind to CXCL12 secreted by astrocytes and microglia. Oligodendrocytes can communicate with microglia through their membrane glycoprotein CD200, which binds with its corresponding receptor on microglia and reduces its activity. Cd200-deficient microglia are spontaneously activated.^[47–51]^ Furthermore, oligodendrocytes also secrete CXC chemokines, like CCL21 and CXCL1-5 in large quantities, which aggravate inflammation. After TBI, OPCs can also release matrix metallopeptidase (MMP)-9, which damages the BBB and promotes neutrophils and monocyte infiltration, thereby, further aggravating inflammation^[52,53]^ (Fig. 3).

## 3. Inflammatory response and neutrophils after TBI

Neuroinflammation is an inflammatory response in the CNS, involving both the brain and peripheral immune cells. Even though neuroinflammation is a response to shield the CNS from injury and infection, it is also a crucial secondary injury mechanism after TBI. TBI-associated neuroinflammation is manifested by stimulated glial cells, astrocytes, recruited white blood cells and up-regulated inflammatory cytokines in the brain center.^[54,55]^ In the CNS, neutrophils are uncommon in the brain parenchyma due to the BBB.^[56]^ Only a small quantity of neutrophils and other immune cells monitors specific compartments such as meninges, CSF, and pia meninges. However, during pathological conditions like infection, ischemia, trauma, and hemorrhage, the number of neutrophils entering the brain tissue increases significantly.^[57,58]^ Zou et al^[59]^ believe that neutrophils replace the role of macrophages after TBI, clearing apoptotic debris and performing post-traumatic repair. MMP9 and MMP13 stimulate neutrophils to rapidly and orderly migrate to the trauma site after TBI. Neutrophil derived cytokines are very complex, except for the common inflammatory cytokines (neutrophil elastase, tumor necrosis factor (TNF) family; Proinflammatory cytokines; CXC-chemokine; CC-chemokines), other anti-inflammatory cytokines, immunomodulatory cytokines, angiogenic factors and neurotrophic factors were also detected in neutrophils.^[60–64]^ In addition to derived cytokines function, neutrophils can also damage the tight connection and permeability of BBB. Neutrophils can decompose the BBB for recruiting more immune cells in response to pathogens, causing tissue damage. Studies have shown that inhibition of activated neutrophils is beneficial to inflammatory regression and subsequent recovery.^[65–71]^ Studies suggest that Maraviroc, TREM2 agonists COG1410, and cordycepin can all play a neuroprotective role by inhibiting neutrophil invasion after TBI.^[22,69,72]^

## 4. Inflammatory response and T lymphocytes after TBI

T cells have a great influence on microglia phenotype and function. After CNS injury, T cells infiltrate the injured tissues. Krämer et al^[73]^ found that systemic reduction of immunosuppressive regulatory T cells (Tregs) causes enhanced T cell infiltration in the injured brain parenchyma through the TBI model of Controlled cortical impact. The presence of T cells in the brain is consistent with increased expression of the interferon-γ (IFN-γ) gene and hyperreactive astrocyte proliferation. Tregs cells depletion diminishes the brain’s acute immune responses. Tregs cells may critically regulate the TBI-associated pathophysiology. The study data of Caplan et al^[74]^ showed that human Treg therapy changed the peripheral and central immune response after TBI in the rodent model and significantly reduced the chronic microglial hyperplasia in the damaged brain hemisphere. Moreover, Treg cells could effectively inhibit the pro-inflammatory immune response of rat spleen and microglial cells. Daglas et al^[75]^ found that activation of CD8^+^ T cells after TBI in mice caused long-term nerve damage. Other studies revealed that IL-15 can stimulate neuronal apoptosis and enhance nerve damage by increasing the CD8 T cells’ function in TBI rat models.^[76]^ Activation of IL-33/ST2 signals can regulate the function of T cells, reduce the size of brain injury, and alleviate functional defects after TBI.^[77]^ Th17 cells, named for their production of IL-17 and other pro-inflammatory cytokines, are thought to be responsible for the demyelination of myelopathy in experimental autoimmune encephalomyelitis.^[78]^ Th17 and other “type 17” T cells are linked with different autoimmune and inflammatory states. The type 17 response can promote and induce the recruitment of CXCL8 and neutrophils by cytokines such as IL-1β released after TBI, thereby exacerbating the inflammatory response.^[79]^ Studies have indicated that umbilical cord mesenchymal stem cells and propofol can assist nerve repair after TBI by modulating Treg/Th17 homeostasis.^[80,81]^ However, T cells are also a double-edged sword for TBI. Studies have found that T cell-deficient mice (due to RAG or MHCII gene defects) have a poor prognosis in CNS injury models, indicating a dominant neuroprotective effect of T cells.^[82,83]^ The activation of mice’s autoimmune T cells is beneficial for the repair of CNS injury, and the underlying mechanism may involve neurotrophic factors produced by T cells acting on neurons and astrocytes to promote survival and repair.^[83–89]^ T cells are necessary for normal CNS development because their deficiency in mice exhibits abnormal cognitive and behavioral development, suggesting they contribute to brain development and maintenance.^[90]^ While regulating M1/M2-like homeostasis, T cells produced IL-4 protects neurons by enhancing neurotrophic signaling. IL-4-mediated T cell protection of damaged CNS tissues does not require T cells’ antigen-specific receptor initiation, and neurons can directly stimulate IL-4.^[82,91]^ IL-33 also has a neuroprotective effect after CNS injury in mice.^[92]^ It acts on IL-4-producing Th2 cells and therefore may be a link between CNS damage and the initiation of IL-4 production.^[93–95]^ The function of T lymphocytes in TBI-mediated recovery and inflammation, and the process of antigen specificity and activation pathway, especially in chronic TBI models, requires further investigations.^[96]^

## 5. The interaction of various cells in the inflammatory response after TBI

### 5.1. Microglia initiation and astrocytes

In vitro investigations have shown that microglial and astrocytes’ interaction is mainly conducted by inflammatory regulators, and microglia-derived cytokines IL-1β, IL-6, TNF, IL-1α, and complement component C1q can inhibit the formation of astrocytes’ intercellular junction and restrict the intercellular traffic. In addition, it can cause loss of astrocytes function and increase astrocytes’ glucose uptake.^[30,97–99]^ However, microglial and astrocytes’ gene expression in an in vitro environment changes rapidly, therefore, it is impossible to reconstruct the in vivo astrocytes and microglial interaction in a dish.^[100–107]^ Recently, Liddelow et al^[108]^ induced ischemia to cause acute CNS injury through injection of lipopolysaccharide or arterial occlusion respectively, and found that activated microglia-stimulated reactive astrocytes, which lost most of their normal cellular functions and produced new neurotoxic functions. They quickly killed neurons and matured differentiated oligodendrocytes. With the help of mice PD model, researchers found that glucagon-like peptide-1 receptor agonists inhibited microglia-mediated transformation of astrocytes into A1 neurotoxic phenotypes and thus played a neuroprotective role.^[109]^ In a mouse experimental autoimmune encephalomyelitis model, microglia-derived transforming growth factor-α reduced the severity of the disease by directly signaling astrocytes.^[110]^ Shinozaki et al^[111]^ demonstrated in a rodent TBI model that inhibiting microglia notably damaged astrocyte scar formation and increased brain injury, indicating the presence of neuroprotective interaction of microglia and astrocyte after TBI.

### 5.2. Microglia initiation and neutrophils

Microglia are the brain’s innate immune cells, similar to macrophages in surrounding tissues. It removes debris, transmits inflammatory signals, and performs other activities. Microglia can be either resting or activated. Their resting state is stimulated by senescence, stroke, brain injury, and neurodegenerative diseases to become an activated state. Currently, no evidence suggests that neutrophils affect microglia subtypes (M1 and M2) in the TBI model. However, Moxon Emre and Schlichter revealed that neutrophils reduction could reduce the number of microglia/macrophages and their promoter marker CD68 after intracerebral hemorrhage, suggesting the involvement of neutrophils in the regulation of microglial status.^[112]^ Kenne et al^[113]^ also demonstrated in TBI mice models that neutrophil depletion decreased the activation of microglia, reduced edema, and loss of brain tissue. After brain injury, microglia stimulate endothelial cell activation and promote peripheral leukocyte recruitment to the CNS.^[114]^ Activated microglia rapidly generate large quantities of inflammatory chemokines (IL-1β, TNF-α, IL-6, CXCL1-5, CXCL8-10) and cytokines. These strong inflammatory regulators recruit and activate neutrophils. Along with the aforementioned microglia-derived cytokines, neutrophils also induce other molecules to mutually initiate microglia, for instance, lipid carrier protein 2, reactive oxygen species, and MMP9.^[13,115–118]^ Activated microglia are beneficial as well as harmful: on 1 hand, it stimulates neutrophils to secrete more pro-inflammatory cytokines, on the other hand, it reduces neuronal damage and the release of microglia neuroprotective factors.^[119]^ However, whether neutrophils affect the M1/M2 polarization of microglia after TBI remains unclear.

### 5.3. Astrocytes and neutrophils

Astrocytes generally have a protective effect during neuroinflammatory processes.^[120]^ Although, reactive astrocytes can form perivascular scarring during acute inflammatory responses, thereby limiting the diffusion of neutrophils from damaged tissue to healthy tissue.^[121]^ in vitro experiments by Xie et al^[122]^ revealed that neutrophils interact with astrocytes in both direct and indirect ways. During direct cell-cell interaction, astrocytes reduce apoptosis and neutrophils’ degranulation and improve the neutrophils’ phagocytosis and the expression of pro-inflammatory cytokines. Indirectly, astrocytes can reduce neutrophil apoptosis, enhance neutrophils’ necrosis and phagocytosis, and inhibit their granulation.^[122]^ Neutrophils and astrocyte communication also affect astrocyte reactivity. Anti-Ly6G antibodies treatment in mice inhibited astrocyte proliferation and worsened the outcome of spinal cord injury.^[123]^ Hooshmand and Nguyen in their in vitro investigation found that neutrophils can promote astrogenesis by producing C1q and C3a.^[124]^ All the above investigations indicate that neutrophils and astrocytes are major sources of cytokines during neuroinflammation, stimulating each other to promote the inflammatory cascade. Neutrophils are important factors in promoting reactive astrocyte proliferation during brain injury. However, whether astrocyte proliferation is beneficial for brain injury is undetermined.

### 5.4. Oligodendrocytes and neutrophils

Oligodendrocytes not only interact with microglia and colloidal astrocytes to participate in immune regulation but also have a certain relationship with neutrophils. During acute brain injury, OPCs are the main component of MMP9-expressing cells. After TBI, OPCs release MMP-9, destroy the BBB, and promote neutrophil infiltration, thereby further aggravating the inflammatory response. Seo et al^[53]^ treated brain-injured mice with MMP inhibitor GM6001 and found that after the second day of surgery, GM6001 reduced early BBB leakage and neutrophil infiltration.

### 5.5. Oligodendrocytes and T lymphocytes

Oligodendrocytes produce a variety of immunomodulators and express multiple receptors, for example, MHC class I molecules expressed by oligodendrocytes are recognized by CD8^+^ T lymphocytes,^[125,126]^ and MHC class II molecules interact with CD4^+^ T lymphocytes. Atypical MHC class I molecules of human leukocyte antigen E were expressed in response to inflammatory cytokine invasion.^[127,128]^ However, whether typical or atypical MHC molecules stimulate T-cell-mediated damage or initiate repair procedures remains to be further investigated. Kaya et al^[129]^ found that oligodendrocytes and microglia cells induced by CD8^+^ T cells and responding to IFN are important ornaments of white matter aging. However, in the TBI model, the exact communication between CD8^+^ T cells and oligodendrocytes, their relation, and their association with IFN signal are still unknown.

## 6. Conclusions and future directions

Neuroinflammatory reactions after TBI include glial cell activation, the brain’s inflammatory regulator’s release, and peripheral immune cell recruitment. These inflammatory reactions not only induce secondary brain injury but also repair the nervous system to a certain extent. Therefore, the post-traumatic neuroinflammatory response has both positive and negative effects. Therefore, future research should focus on clarifying the specific roles of various inflammatory cells and factors and finding the corresponding inflammatory regulation strategies over time after TBI, to strategize the cure according to these different times and its associated factors, which play an important role in promoting nerve repair and regeneration while reducing the secondary damage of inflammatory response. Clinical treatment cannot simply involve the suppression of inflammatory response, the inflammatory phenotype of each patient based on their sex, genetic predisposition, age, presence or absence of secondary damage, CSF, serum, imaging, and biomarkers should also be defined. Clinical trials of patient-specific personalized inflammatory regulation therapy are expected to reduce secondary damage, enhance repair and improve patient outcomes.

## Author contributions

**Formal analysis:** Jianhua Zhang.

**Software:** Hongru Li.

**Writing – original draft:** Huige Li.

**Writing – review & editing:** Qinghui Zhao, Fei Xie.
